# Supplementary material for: Transcriptomic and Metabolomic Analyses Providing Insights into the Coloring Mechanism of Docynia delavayi
Source: Foods. 2022 Sep 18;11(18):2899. doi: 10.3390/foods11182899 (PMC9498648; doi:10.3390/foods11182899)
Supplement: Supplementary file 1 [file foods-11-02899-s001.zip › Figures supplement.pdf]

Transcriptomic and metabolomic analyses providing insights into the Coloring Mechanism of *Docynia delavayi*

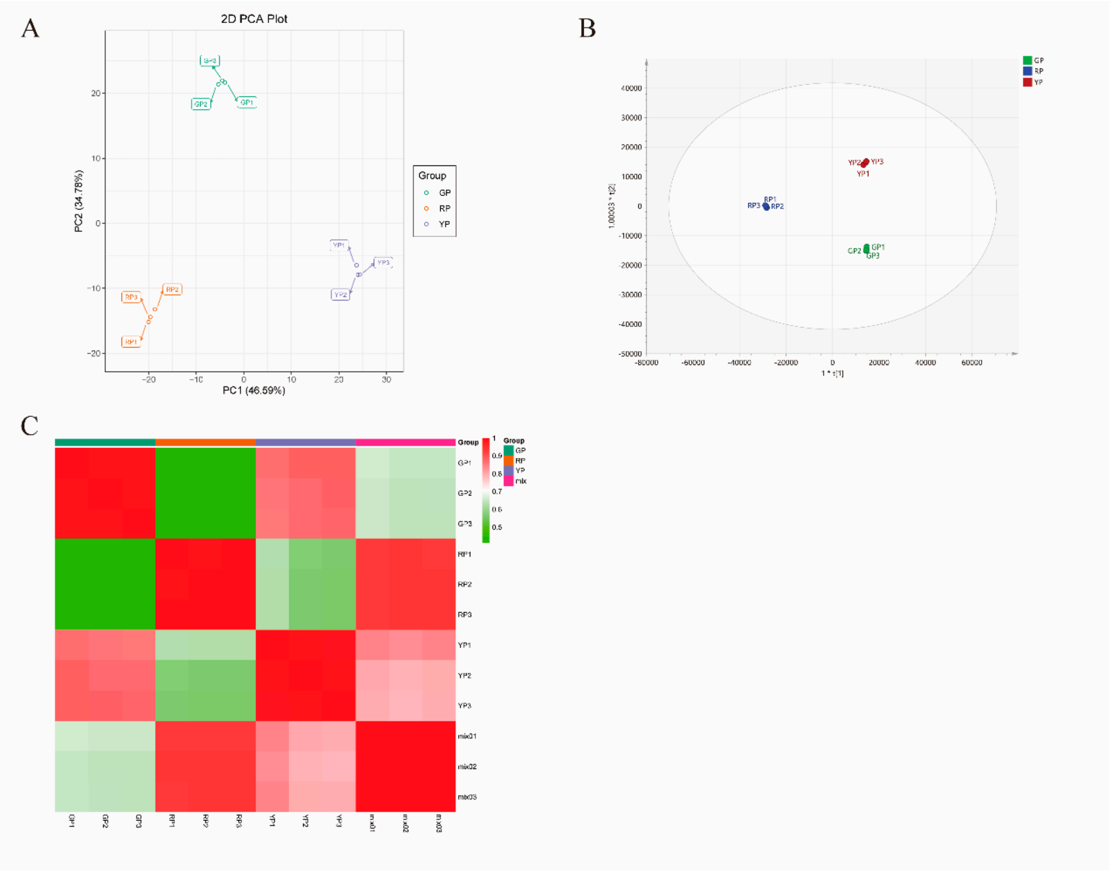

Figure S1 Metabolite samples (GP, YP, and RP) quality assurance. (A) Principal component analysis (PCA) of metabolite data. (B) OPLS-DA score plot. (C) Heat map of Pearson's correlation coefficient for metabolite samples.

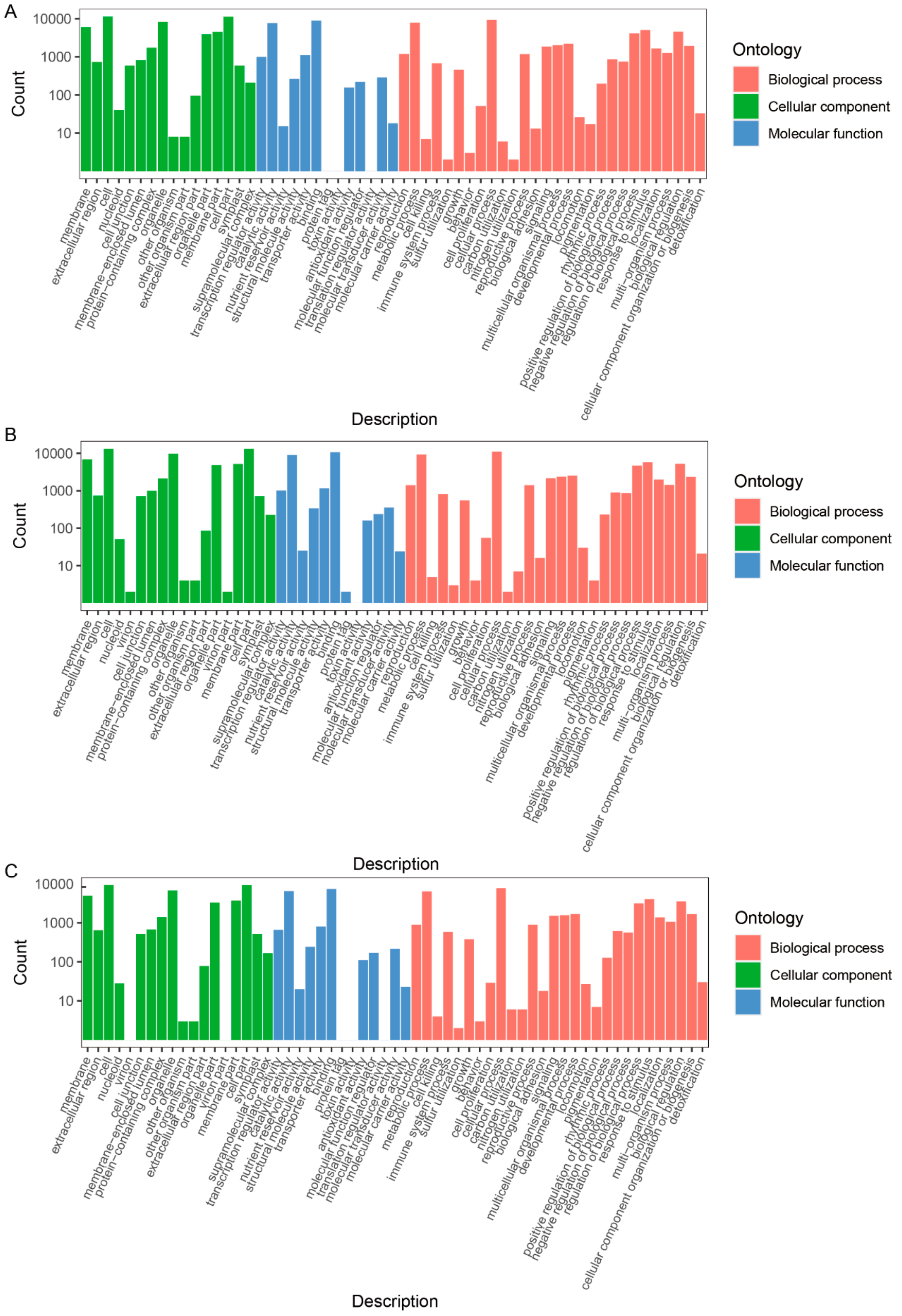

Figure S2 DEGs analyzed by GO enrichment (A) YP vs. GP, (B) YP vs. RP, and (C) GP vs. RP.

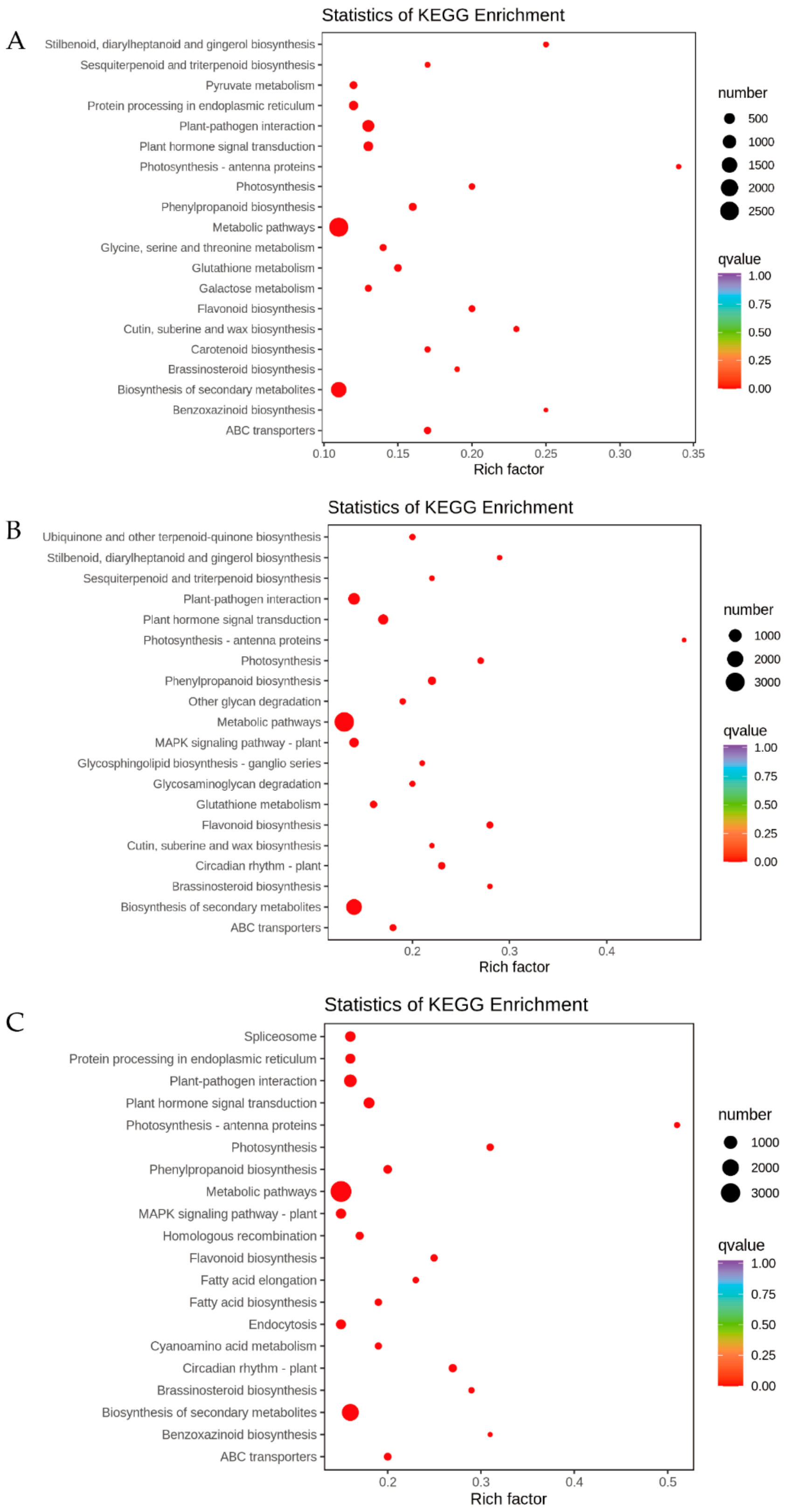

Figure S3 DEGs analyzed by KEGG enrichment analysis (A) GP vs. RP, (B) YP vs. GP, and (C) YP vs. RP.
